# Clinical Cases

**Published:** 1895-10

**Authors:** Olin M. Drake

**Affiliations:** Boston, Mass.


### CLINICAL CASES.

Olin M. Drake, M. D., Boston, Mass.

### Case I.—Tumor of the Neck.

Mrs. S., of South Brooksville, Me., consulted me for the first time on the 20th of March, 1889, for a tumor that she had on the right side of her neck. The centre of the tumor was about over the angle of the jaw; in size it was somewhat larger than a good-sized fist. It was perfectly solid (*very hard*) and very firmly attached (it could not be moved one iota without moving the whole head), and in such a manner that the motion of the jaw was very much impeded. The surface was not smooth, neither was it nodular, but uneven. Its outline was rather ill-defined, the skin over it was firmly attached with many enlarged cutaneous veins, and its color was deep reddish-purple; it looked precisely the same as I have observed in cancer of the breast just preceding ulceration or breaking down of the tissues. I was unable to find enlarged glands elsewhere. She gave me the following history, and I will give it as she gave it to me, in her own words:

"In April, 1888, a grandchild of mine of four years received a kick from a horse, from which he died in the early part of the following July, and during the time I took the whole care of him, both night and day. About the middle of May there appeared a little bunch; when first noticed it was the size of a pea, and it gradually increased. As we had several physicians attending the child during its sickness, I consulted them to find out what this little bunch could be. One pronounced it scrofula, and gave me Tincture of Iodine for a wash, but it did no good. Another said it was an enlarged gland, and advised my washing it with Soda water, which I did for about one week, and I think it went nearly away. This was about the first of July. I did not think much more about it till the last part of December, when it began to increase in size very rapidly, and before very long I was unable to lie on my right side on account of its size. During this time I received the attention of two or three physicians, and the last part of February they concluded that I must undergo a surgical operation and have the tumor removed, and for that purpose I came here (Ellsworth) and consulted Drs. Manning and Hagerthy, and they concluded to remove it, and appointed the day for the operation, but the next morning Dr. Manning visited me and told me that the operation was too severe for them to undertake, and advised my going to Portland to the Maine General Hospital, and I was taken to that institution in a few days and was examined by the surgical staff. Each one examined me separately, and after their consultation the head surgeon informed me that my trouble was cancer, and that nothing could be done in the way of an operation on account of its being so firmly and deeply attached, and involving the important blood-vessels, making it impossible to remove it entire, and to remove it in part would cause it to spread or extend itself much faster than if let alone. They advised my returning home, and cautioned me to let it most severely alone and allow no one to have anything to do with it. And now, Doctor, this is all I can tell you; I know my fate, and I am resigned; but is there not something that can be done for these pains? I know Mrs. H. had no sufferings under your treatment. I know nothing about your mode of treatment, but I'll assure you that I will be a most dutiful and faithful patient if you can only relieve me and give me sleep."

The scarcity of subjective symptoms in her case was remarkable; besides the objective symptoms given she complained of no appetite, extreme sluggishness of the bowels and general prostration, but her great complaint was her inability to sleep and the pains in the tumor. This was the way she put it: "The moment I try to go to sleep the pains will begin in the tumor; first it will be a *boring sensation all through the tumor*, followed by *sharp darting pains*." I promised her nothing, but said I would see what could be done. I put up sixteen powders of Conium$^{200}$ (Dunham), and directed her to put a powder into water each day and take two teaspoonfuls every two hours. This was March 20th, 1889. On April 6th I got my first report, and there was no change whatever. I sent her twelve powders of sugar of milk, to be used in the same way. On April 19th there was no change, and I sent three powders of Conium$^{500}$ (Tafel), and nine of Sac-lac., and on May 2d my third report, but there was no change. What should I do? I could not make a better prescription, and my rule is "*when in doubt, don't*," so I sent Sac-lac., and not until May 28th did I get the first encouraging word, and in this report she says: "Doctor, I think, if anything, I have a little less pain and of course a little more sleep, and I am eating a little more of late." *I never gave her another dose of medicine*, though she continued to take Sac-lac. till the 1st of October. June 22d she reported improvement, and stated "if she was not very much mistaken the tumor was not so hard and was softer." It kept on decreasing in size till September 21st, when she informed me it was all gone. I had the pleasure of examining her personally in December, and I could find nothing of it, neither was I able to obtain from her one abnormal symptom, subjective or objective, in fact, so far as I was able to find out, she was a perfectly healthy old lady of seventy years.

### Case II.—Imaginary Eating.

In 1890 I was called to see an old man, a sufferer of dementia senilis; he had not slept for three nights, requiring the constant attendance of two people to keep him on the bed; face very red, eyes injected and wild, talkative, inclined to bite, and all sorts of illusions. The most marked symptom in his case was *imaginary eating;* he would see different articles of food here and there in the room, and would try to get out of bed for them, or he would pick what he would say was a very choice piece of cake from his coverlet, and after taking a bite of it he would then hold it off at arm's length, and make his comments upon its good qualities; and thus he had been eating for three nights and days. He would masticate and swallow as though it was all a reality. Now I had never met this symptom, and did not know the remedy, but I did what I thought was right. I gave one dose of Dunham's 200th of Bell., and put another dose into water, for him to take a teaspoonful every half-hour till they should report at my office at six o'clock. (I gave him the dose on the tongue at 4 P. M.) By that time I should have an opportunity of looking up the case, and I found the symptom given by Allen under Atropinum. I was then more interested than ever. Atropinum, one of the active principles of Belladonna, had produced the symptoms; would Belladonna remove it? This case was in a family in which they differed very strongly in their medical ideas; one portion of them insisted that Dr. H. should be called, for then they could have Morphine given, which they knew I would not give. But to return to our case. At six o'clock the son came to report, and this was what he said: "Well, Doctor, it did it!" "Did what?" I asked. "Why, put father to sleep, and we can't wake him up to give him any of the medicine in the glass." And thus it was, inside of ten minutes after taking the dry dose he went to sleep, and he *slept through that night, all the next day, and all the next night* till after five o'clock in the morning, and when he awoke he told his wife that he was feeling perfectly comfortable and wanted some nourishment. But I got the credit of being a dangerous prescriber of Opium from that faction of the family that did not want me called in in the first place, and probably they could not be convinced to the contrary. The above patient died two or three months since. I continued to prescribe for him off and on. Once only afterward did this symptom return, but in a much less marked degree and I tried Atropinum (Tafel's 200th), but under its action for twenty-four hours it seemed to increase, and I gave him Belladonna$^{40m}$ (Fincke), and it disappeared for good.

